# Donation initiators: Shifting the focus from outcome to first‐time engagement ‐ An interview study

**DOI:** 10.1111/trf.18365

**Published:** 2025-08-06

**Authors:** Klara Greffin, Melissa J. Völter, Fanni Peters, Thomas Thiele, Barbara M. Masser

**Affiliations:** ^1^ Institute of Psychology University of Greifswald Greifswald Germany; ^2^ School of Psychology The University of Queensland St Lucia Queensland Australia; ^3^ Institute of Transfusion Medicine University Medicine Greifswald Greifswald Germany

**Keywords:** blood donation, deferral, donation initiators, qualitative study

## Abstract

**Background:**

A stable whole‐blood supply is vital to healthcare and relies on the successful recruitment and retention of donors, with first‐time donors playing a key role in replacing those who lapse over time. The initial donation attempt is emotionally and cognitively significant, and deferral at this stage can lead to disappointment and disengagement. Language that foineligibility rather than intent may reinforce negative self‐perceptions and reduce the likelihood of future donation.

**Objective:**

This study aimed to explore the subjective experiences of deferral among first‐time donation candidates and to identify a term that accurately reflects their intent to donate.

**Methods:**

Twenty individuals temporarily deferred during their first whole‐blood donation attempt participated in semi‐structured interviews. A constructivist‐phenomenological approach guided data collection and analysis, focusing on participants' motivations, identity perceptions, and preferences for appropriate terminology. Suggested terms were evaluated through qualitative content analysis based on four criteria: donation status and context, avoidance of donor labeling, positive and inclusive language, and bilingual feasibility.

**Results:**

Fifteen out of 20 participants did not see themselves as blood donors due to the absence of a completed donation. They proposed 25 terms, grouped into 17 categories. “Donation Initiators” emerged as the preferred label, positively acknowledging the intent without implying failure and framing deferral as part of the donation journey.

**Conclusion:**

Introducing the term “Donation Initiators” offers a low‐threshold, linguistically inclusive alternative that may improve communication, reduce negative emotional reactions to deferral, and support donor engagement. Future research should investigate its impact on donor identity and retention.

## 1 BACKGROUND

A reliable whole‐blood supply is the backbone of modern healthcare^1^ and requires continuous efforts to both recruit and retain donors. Recruiting first‐time donors is crucial in offsetting the natural attrition of retained donors that occurs due to age, health changes, or other life circumstances.^2^, ^3^, ^4^

The first donation attempt marks a particularly vulnerable step in a donor's journey.^5^ Drawing on the Transtheoretical Model of Behavior Change (TTM),^6^ this first attempt moves the individual from the contemplation stage, where they assess the benefits, feasibility, and personal implications of donating, through preparation to donate to the action stage, where they commit to the donation process. However, in the context of blood donation, this transition involves not only cognitive adjustments but also affective and emotional changes. Many potential donors enter donation centers brimming with genuine joy and anticipation,^7^ eager to make a positive impact.

Yet not everyone who attempts to donate blood is eligible. Eligibility is assessed either in advance through pre‐registration or on‐site before donation. When donors do not meet these criteria, deferrals are necessary to protect donor health and/or recipient safety.^8^, ^9^ A deferral at this crucial moment can lead to a notable drop in mood,^10^ putting these attendees on an emotional trajectory to permanent lapse.^7^ Accordingly, being deferred during an initial donation attempt significantly reduces the likelihood of returning to donate once eligible, as shown by previous research.^5^, ^11^

Beyond the immediate emotional and behavioral impact of deferral, the language used may shape the attendees' cognitive understanding of the experience, which in turn may reinforce negative emotions and reduce the likelihood of return. Healy et al.^12^ observed that stigmatizing language in healthcare can lead to feelings of exclusion, rejection, and self‐doubt, ultimately reducing engagement. Similarly, the language used in deferral can significantly affect self‐perception and emotional response, further compounding the challenges of re‐engagement.^13^ In the context of blood donation, labeling deferred individuals in a way that emphasizes ineligibility rather than intent to donate (e.g., failed donor) risks reinforcing a sense of blame or inadequacy. This, in turn, may make them less likely to attempt donating again, further exacerbating the challenge of donor retention. However, even more neutral terms like “first‐time donor” are overly broad, as they fail to differentiate between those who donate and those who are deferred ‐ with the latter, strictly speaking, not being donors.

Changing a label is a potentially simple yet effective intervention that can positively impact donor retention. Therefore, adopting more inclusive and precise terminology that is more supportive and shifts the focus from deferral outcomes to donation intent may maintain the motivation and engagement of this promising group of not‐yet donors ‐ arguably our most viable prospects for future donor recruitment. Consequently, this study aims to determine an appropriate and inclusive term for individuals who experience a deferral during their first blood donation attempt.

## 2 METHODS

### 2.1 Research design

This exploratory qualitative study examined how individuals temporarily deferred during their first attempt to donate whole blood experienced the deferral and which terms they felt best described their situation. Using a constructivist, phenomenological approach, semi‐structured individual interviews were conducted by a trained psychologist. Reflexivity was maintained throughout to minimize bias and acknowledge the researchers' potential influence on data collection and interpretation.

### 2.2 Participants and recruitment

Twenty^14^ potential first‐time whole‐blood donors who were temporarily deferred during their initial attempt were recruited from the University Medicine Greifswald Blood Donation Centre. Individuals with prior donation experience or permanent deferrals were excluded. Recruitment ceased upon reaching data saturation.

Participants completed a standard donor screening process. Deferred individuals were referred to a transfusion physician, who explained the deferral and invited them to participate in the study. Informed consent was obtained from all participants, with interviews conducted on‐site immediately following the deferral. Interviews lasted 16 minutes on average.

### 2.3 Data analysis

Interviews were transcribed verbatim and analysed using a simplified version of qualitative content analysis.^15^ The data analysis followed the structure of the interview guide, focusing on the motivations behind attempting to donate, experiences with deferral, identification as a blood donor, and the selection of a suitable term for individuals deferred during their first blood donation attempt. The evaluation of proposed terms was guided by four criteria, developed through a combination of researcher‐driven and participant‐informed insights:

- *Donation Status and Context*: Highlighting the individual's initiation of the donation process.
- *Avoidance of Donor Labeling*: Ensuring accurate representation of those who have not yet donated.
- *Positive and Inclusive Language*: Promoting a supportive, non‐judgmental tone.
- *Bilingual Feasibility*: Ensuring usability in both English and German contexts.

## 3 RESULTS

### 3.1 Participants

Twenty participants were included. Among them, 12 identified as women and 8 identified as men. Participants had a mean age of 22.5 years (*SD* = 4.95). Educational backgrounds included secondary school diplomas (*n* = 5) and university entrance qualifications (*n* = 15). Motivations for donating included altruism and encouragement from family or friends, while common reasons for deferral were hemoglobin deficiency (*n* = 6) and incomplete or invalid documentation (*n* = 5).

### 3.2 Donor identity

When asked whether they identified as blood donors, 15 (75%) participants responded negatively, explaining that they believed a successful donation was a prerequisite for such identification. Three participants identified as donors based on prior donations for research (*n* = 1) or their sense of contribution (*n* = 2). Two participants expressed alignment with donor values but did not view themselves as donors yet (Table 1).

**TABLE 1:** Descriptive overview of the sample.

Characteristic	*n*	Mean (*M*) + standard deviation (*SD*)	Percentage (%)
Age		22.5 years ± 4.95 years	
Sex			
Male	8		40
Female	12		60
Educational level			
Secondary school certificate	5		25
University entrance qualification	15		75
*Motivators for first blood donation attempt* ^a^			
Altruism	16		
Social influence	11		
Donating blood is important	10		
Known need for blood	7		
Reciprocity^b^	6		
Low personal effort or lack of barriers	4		
Personal values	4		
Curiosity	4		
Planned action	3		
Social responsibility	3		
Health check‐up	2		
Remuneration	2		
Miscellaneous	4		
*Reasons for deferral*			
Hemoglobin deficiency	6		30
Identity documents	5		25
Stay in Malaria area	2		10
New tattoo	1		5
New piercing	1		5
Recent infection with fever	1		5
Pending multiple sclerosis diagnosis and medication	1		5
Medication	1		5
Recent vaccination	1		5
Low body weight	1		5

^a^ Participants could state multiple motivators.

^b^ Motivated by the idea that they may need a blood transfusion in the future.

### 3.3 Suggested terminology

Participants proposed 25 terms, grouped into 17 categories (Table 2). The term “Donation Initiators” emerged as the most suitable, meeting all criteria while resonating with participants' feedback.

**TABLE 2:** Suggested terms for ineligible first‐time blood donors.

German term^a^	English translation	Donation status and context	Avoidance of donor labeling	Positive and inclusive language	Grammatically correct and bilingual feasibility
Blutspender*innen	Blood Donors	ꭓ	ꭓ	✓	✓
Spendeinitiator*innen	Donation Initiators	✓	✓	✓	✓
Angehende Blutspender*innen	Prospective Blood Donor	?	ꭓ	✓	✓
Blutspendeversucher*innen	Blood Donation Attempters	✓	✓	✓	ꭓ
Blutspendewillige*r	Person willing to donate Blood	ꭓ	✓	✓	ꭓ
Blutspendebereit	Ready to Donate Blood	ꭓ	✓	✓	✓
Bemühte Blutspender*innen	Dedicated Blood Donors	ꭓ	ꭓ	✓	✓
Sich im Prozess befindend	Currently in Process	ꭓ	✓	✓	ꭓ
Blutspendebefürworter*innen	Blood Donation Advocates	ꭓ	✓	✓	ꭓ
Passive Blutspender*innen	Passive Blood Donors	ꭓ	ꭓ	?	✓
Möchtegern‐Blutspender*innen	Wannabe Blood Donors	ꭓ	ꭓ	ꭓ	ꭓ
Wunschspende	Hoped‐for Donation	ꭓ	✓	✓	ꭓ
Nicht‐Spende‐Person	Non‐Donation‐Person	ꭓ	✓	ꭓ	ꭓ
Nicht spendeberechtigte Person	Person ineligible for Donation	ꭓ	✓	ꭓ	✓
Person mit einer Fehlspende	Person with a Failed Donation	ꭓ	✓	ꭓ	ꭓ
Abgelehnte*r Erstblutspender*in	Rejected First‐Time Blood Donor	ꭓ	ꭓ	ꭓ	✓
Guter Wille, aber nicht hinbekommen	Good intentions, but did not manage to	ꭓ	✓	ꭓ	ꭓ

*Note*: ✓ means that the aspect is present, *ꭓ* means that the aspect is (rather) absent, ? means unclear or open to discussion.

^a^ As the interviews were conducted in German, the original responses are included alongside the translation in the interest of transparency.

## 4 DISCUSSION

Traditional labels often fail to capture the intentions and contributions of individuals who are temporarily deferred during their first donation attempt. In our study, we explored the experiences of these individuals and found that most did not identify as blood donors, believing that a successful donation was necessary to warrant such a label. This misalignment underscores the inadequacy of conventional terms like “deferred first‐time donor.” In response, participants proposed several alternatives, with “Donation Initiators” emerging as the most appropriate.

### 4.1 Who are Donation Initiators?

Donation Initiators are individuals who are temporarily deferred at their first attempt to donate. This term recognises the proactive effort of these individuals in starting their journey as a donor, distinguishes them from donors or non‐donors who have not attempted to donate, while avoiding attributions of failure or blame. It reframes deferral as a step in the donation journey, focusing on the action rather than the outcome. By avoiding negative labeling, “Donation Initiators” reinforces the accomplishment of moving to action. Further, the ongoing focus on intent rather than (failed) outcome may encourage future participation.

### 4.2 Implications and limitations

First‐time donors are particularly vulnerable in the blood donation continuum, with deferrals during their initial attempt significantly reducing the likelihood of future donations.^5^, ^11^ The mechanisms underpinning these adverse effects are not well understood, necessitating an examination of both donor‐specific factors and institutional practices at blood donation centres. Adopting “Donation Initiators” represents a strategic shift toward more inclusive and supportive communication. This reframing acknowledges the donor's intent and effort, integrating deferral into the donation process as a normative, rather than stigmatizing, event. Using inclusive terminology, especially when paired with less negative terms for deferral (e.g., postponement^16^), can help mitigate the negative emotional responses often triggered by deferral, resulting in a more positive donor experience. Future research should test whether this change in labeling can improve the emotional responses and self‐perception of these deferred individuals.

While the current research is limited to German‐speaking participants, recruited in a regional context, the introduction of “Donation Initiators” provides an important refinement of deferral categorisation for first‐time donors. Future investigations should establish the generalisability of this term, and also examine whether consistent use can facilitate the formation of a positive donor identity. Clearly defining this group helps identify Donation Initiators by their demographics, motivations, deferral reasons, and retention patterns ‐ distinguishing them from deferred experienced donors. These insights are key to addressing their needs and designing interventions that foster long‐term engagement.

## 5 CONCLUSION

The introduction of “Donation Initiators” marks a step toward more inclusive and supportive communication in the blood donation context. By focusing on effort and intent, this term reframes deferral as a normal and accepted part of the donation journey, encouraging deferred individuals to return and try again. Implementing and testing the effects of this term might improve the donor experience, retention efforts, and ultimately, the blood supply chain.

## FUNDING INFORMATION

This work was supported by start‐up funding for early‐career researchers provided by the University of Greifswald.

## CONFLICT OF INTEREST STATEMENT

The authors declare that they have no conflict of interest.

## ETHICS STATEMENT

The study received ethical approval from the institutional review board of the University Medicine Greifswald (BB 118/23), and all participants provided informed consent.
